# Smoking during pregnancy. Results of the cross-sectional KiGGS Wave 2 study and trends

**DOI:** 10.17886/RKI-GBE-2018-026

**Published:** 2018-03-15

**Authors:** Benjamin Kuntz, Johannes Zeiher, Anne Starker, Franziska Prütz, Thomas Lampert

**Affiliations:** Robert Koch Institute, Berlin, Department of Epidemiology and Health Monitoring

**Keywords:** MATERNAL SMOKING, TOBACCO, PREGNANCY, HEALTH MONITORING, KIGGS

## Abstract

Maternal smoking during pregnancy poses a significant risk to the development of unborn children. Data from KiGGS Wave 2 shows that 10.9% of mothers of 0 to 6 year-old children smoked during pregnancy. Mothers who were under 25 when giving birth smoked about two to three times more often than older mothers. Furthermore, there is a distinct social gradient in maternal smoking: a higher socioeconomic status is associated with a lower proportion of children with a mother who smoked during pregnancy. A comparison with data from the KiGGS baseline study shows that the proportion of mothers who smoked during pregnancy fell from to 19.9% to 10.9% between the two study periods. Thus, the KiGGS results are in line with those from the perinatal survey, which also found that the proportion of pregnant women who smoke has declined significantly since the mid-1990s.

## Background

Maternal smoking during pregnancy poses a significant risk to the development of an unborn child [1-3]. Complications during pregnancy such as miscarriages, premature births and stillbirths occur more frequently among women who smoke. The harmful substances contained in tobacco smoke pass through the placenta into the bloodstream of unborn children and impair the supply of oxygen, thus inhibiting growth and essential processes of fetal maturation. Therefore, babies born to mothers who smoke are, on average, both smaller and lighter and have a smaller head circumference at birth than babies born to non-smokers [4]. Maternal smoking during pregnancy also promotes the development of congenital malformations [5] and is a major risk factor linked to sudden infant death syndrome [6]. It also increases the long-term risk of numerous diseases and developmental disorders in childhood, including asthma [7], otitis media [8], overweight [9] and behavioural problems [10].

Mothers who stop smoking before or during pregnancy can significantly reduce their risk of complications and of adverse health effects for both mother and child [11]. As such, tobacco prevention, cessation and control among pregnant women and women of childbearing age are high priorities from a public health point of view [12]. The health target ‘Reduction of tobacco consumption’, which was developed as part of the process to develop national health targets in Germany and revised in 2015, includes one out of five sub-goals that aims at reducing maternal smoking rates during pregnancy [13]. The health target ‘Health before, during and after birth’, which was adopted in 2017, additionally aims to reduce the numbers of women who smoke during pregnancy [14]. In order to monitor how well these goals are being achieved, repeated epidemiological studies of the spread of tobacco use among pregnant women are needed. This is the only way to identify risk groups, develop suitable measures for the reduction of maternal smoking during pregnancy and evaluate the effectiveness of such measures [3]. The latest German Health Interview and Examination Survey for Children and Adolescents (KiGGS Wave 2) provides data that can be used for this purpose.

**KiGGS Wave 2**

Second follow-up to the German Health Interview and Examination Survey for Children and Adolescents

**Data owner:** Robert Koch Institute

**Aim:** Providing reliable information on health status, health-related behaviour, living conditions, protective and risk factors, and health care among children, adolescents and young adults living in Germany, with the possibility of trend and longitudinal analyses

**Study design**: Combined cross-sectional and cohort study

**Cross-sectional study in KiGGS Wave 2**

**Age range:** 0-17 years

**Population:** Children and adolescents with permanent residence in Germany

**Sampling:** Samples from official residency registries - randomly selected children and adolescents from the 167 cities and municipalities covered by the KiGGS baseline study

**Sample size:** 15,023 participants

**KiGGS cohort study in KiGGS Wave 2**

**Age range:** 10-31 years

**Sampling:** Re-invitation of everyone who took part in the KiGGS baseline study and who was willing to participate in a follow-up

**Sample size:** 10,853 participants

**KiGGS survey waves**

► KiGGS baseline study (2003-2006), examination and interview survey
► KiGGS Wave 1 (2009-2012), interview survey
► KiGGS Wave 2 (2014-2017), examination and interview survey

More information is available at www.kiggs-studie.de/english

## Indicator and methodology

KiGGS forms part of the health monitoring programme undertaken at the Robert Koch Institute and includes repeated cross-sectional surveys of children and adolescents aged between 0 and 17 years that are representative of the German population (KiGGS cross-sectional study). After having carried out the baseline study as an interview and examination survey between 2003 and 2006, and KiGGS Wave 1 as an interview-based survey between 2009 and 2012, KiGGS Wave 2 was implemented between 2014 and 2017 as a combined interview and examination survey. A detailed description of the methodology used in KiGGS Wave 2 can be found in New data for action. Data collection for KiGGS Wave 2 has been completed in issue S3/2017 as well as KiGGS Wave 2 cross-sectional study – participant acquisition, response rates and representativeness in issue 1/2018 of the Journal of Health Monitoring [15, 16].

Data on maternal smoking during pregnancy was recorded retrospectively for KiGGS Wave 2 using information provided by a child’s parents or guardians as part of a written questionnaire. This included asking the question, ‘Did the mother of the child smoke during pregnancy?’. The response categories were ‘Yes, regularly,’ ‘Yes, sometimes’ and ‘No, never’; the first two categories are combined below [3].

The findings presented here are based on data from 4,838 children aged 0 to 6 with valid data on maternal smoking habits during pregnancy. The results are presented as prevalences (frequencies) and are stratified by age of the mother at the time the child was born [3], socioeconomic status (SES) of the family [17] and migration background [18]. Comparable data from the KiGGS baseline study are used to analyse trends over time.

The calculations were carried out using a weighting factor that corrects for deviations within the sample from the population structure with regard to age in years, gender, federal state, nationality and the parents’ level of education (Microcensus 2013 [19]).

This article reports prevalences with 95% confidence intervals (95% CI). A statistically significant difference between groups is assumed to have been demonstrated with p-values of less than 0.05 (once weighting had been applied and the survey design had been taken into account).

## Results and discussion

Data from KiGGS Wave 2 demonstrate that 10.9% of mothers of children aged between 0 and 6 and born between 2007 and 2016 smoked during pregnancy. Mothers who were under 25 when they gave birth had a 22.5% prevalence of smoking during pregnancy; this was around twice as high as the prevalence identified among women who gave birth between 25 and 29 years of age. The proportion of women who smoked during pregnancy was about three times higher in mothers under 25 than among mothers who were 30 or above when they gave birth (Table 1). In addition, a clear social gradient could be observed: the higher the socioeconomic status (SES) of a family, the lower the proportion of children with a mother who smoked during pregnancy (Table 1). Whereas more than one in four children (27.2%) from the low SES group were exposed to tobacco smoke due to maternal smoking during pregnancy, this applied to just one in eleven children (9.2%) from the medium SES group and very few children (1.6%) from the high SES group. Whereas 12.2% of children with no recent family history of migration were exposed to maternal smoking during pregnancy, children with a one-sided migration background were slightly less (9.6%), and children with a two-sided migration background were much less affected (6.2%) (Table 1).

The results from KiGGS Wave 2 are consistent with the findings from the two previous KiGGS waves; other studies also show that smoking during pregnancy is particularly common among certain risk groups [3, 20-23]. These risk groups include mothers who are relatively young when they give birth and socially disadvantaged women. The fact that mothers of children with a two-sided migration background smoked less often during pregnancy was demonstrated by the KiGGS baseline study and KiGGS Wave 1 after taking into account the facts that these families usually face worse social conditions and that mothers in this group are usually younger when they give birth [3].

**Figure 1:**
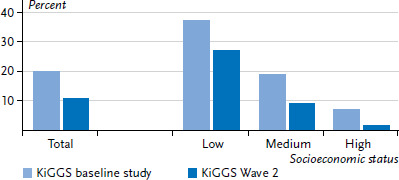
Trends in smoking behaviour during pregnancy among mothers of 0 to 6 year-old children in total and according to their socioeconomic status Source: KiGGS baseline study (2003-2006, birth cohorts 1996-2006, n=6,525) and KiGGS Wave 2 (2014-2017, birth cohorts 2007-2016, n=4,838)

A comparison of the data on maternal smoking during pregnancy from KiGGS Wave 2 (0 to 6 year-old children; 2007-2016 birth cohorts) with corresponding data from the KiGGS baseline study (0 to 6 year-old children; 1996-2006 birth cohorts) demonstrated that the proportion of mothers who smoke during pregnancy has fallen from 19.9% to 10.9% (Figure 1). Although the data also point to a decrease in the prevalence of smoking among mothers with higher SES as well as among those from disadvantaged groups, the existing pronounced social inequalities in maternal smoking behaviour during pregnancy have remained largely stable. The German Perinatal Survey also found that the proportion of pregnant women who smoke has declined since the mid-1990s [24]. The study, which is undertaken as part of external inpatient quality assurance, gathers data on cigarette smoking during pregnancy from all women who give birth in German hospitals. Scholz et al. use this data to demonstrate that the proportion of pregnant women who smoke decreased from 23.5% to 11.2% between the period ranging from 1995 to 1997 and from 2007 to 2011 [24]. Although data from international studies from many countries indicates a decline in prevalence over the past 10 to 20 years, they also demonstrate that a significant proportion of women continue to smoke during pregnancy in most Western countries [25, 26]. The 2013 European Perinatal Health Report also indicates that the proportion of mothers who smoke during pregnancy decreased between 2004 and 2010 in countries such as the UK, France and the Netherlands [27].

However, a number of limitations need to be taken into account when analysing the KiGGS data [3]. On the one hand, the results presented here cannot be compared directly with interview surveys conducted with pregnant women. KiGGS collected its data on maternal smoking during pregnancy retrospectively from parents of 0 to 6 year-old children. By the time the mothers participated in KiGGS, up to six years had elapsed since their pregnancy; as such the information they supplied could be affected by recall bias. On the other hand, the well-known phenomenon of participants’ providing what they view to be socially acceptable responses could also have led to an underestimation of the actual proportion of smokers within the data (social desirability bias). Moreover, the data from KiGGS Wave 1 were not taken into account in the results presented here for methodological reasons, since the birth cohorts (2002-2012; 0 to 6 year-olds) that participated in KiGGS Wave 1 largely overlap with those of the KiGGS baseline survey and that of KiGGS Wave 2. For the sake of completeness, however, it should be noted that KiGGS Wave 1 found that 12.0% of mothers smoked during pregnancy, which is slightly higher than the figures identified from KiGGS Wave 2.

Despite these limitations, the KiGGS data on maternal smoking during pregnancy provide valuable information for epidemiological research and health policy-making. The cross-sectional data, and, in particular, the data from the KiGGS cohort [15, 28], can be used to examine both short-term and long-term links between maternal tobacco use in pregnancy and a child’s health development. Future target group-specific tobacco prevention and cessation measures should increasingly focus on young and socially disadvantaged women. Given that pregnancy provides a window of opportunity for measures that can encourage people to change their behaviour [2], midwives, doctors and other professionals who regularly work with pregnant women should ask mothers about their use of tobacco, educate smokers about the risks, and recommend to quit smoking; where appropriate, support services should be offered [29].

## Key statements

- The proportion of mothers who smoked during pregnancy dropped from 19.9% to 10.9% between the KiGGS baseline study and KiGGS Wave 2.
- Mothers who were under 25 when they gave birth smoked about two to three times more often during pregnancy than older mothers.
- The higher the socioeconomic status, the lower the proportion of children whose mother smoked during pregnancy.

## Figures and Tables

**Table 1:** Prevalence of maternal smoking during pregnancy according to the mother’s age when giving birth, socioeconomic status and migration background Source: KiGGS Wave 2 (2014-2017), children aged between 0 and 6 (n=4,838)

	%	(95% CI)
**Mother’s age when giving birth**		
<25	22.5	(17.5-28.5)
25-29	12.7	(10.4-15.4)
30-34	7.4	(5.9-9.3)
≥35	7.6	(5.7-10.0)
**Socioeconomic status**		
Low	27.2	(22.8-32.1)
Medium	9.2	(7.8-10.9)
High	1.6	(0.9-2.9)
**Migration background**		
None	12.2	(10.5-14.0)
One-sided	9.6	(6.4-14.4)
Two-sided	6.2	(4.2-9.0)
**Total**	**10.9**	**(9.6-12.4)**

CI=confidence interval
